# The effects of dissipation on topological mechanical systems

**DOI:** 10.1038/srep32572

**Published:** 2016-09-08

**Authors:** Ye Xiong, Tianxiang Wang, Peiqing Tong

**Affiliations:** 1 Department of Physics and Institute of Theoretical Physics, Nanjing Normal University, Nanjing 210023, P. R. China; 2 Jiangsu Key Laboratory for Numerical Simulation of Large Scale Complex Systems, Nanjing Normal University, Nanjing 210023, P. R. China

## Abstract

We theoretically study the effects of isotropic dissipation in a topological mechanical system which is an analogue of Chern insulator in mechanical vibrational lattice. The global gauge invariance is still conserved in this system albeit it is destroyed by the dissipation in the quantum counterpart. The chiral edge states in this system are therefore robust against strong dissipation. The dissipation also causes a dispersion of damping for the eigenstates. It will modify the equation of motion of a wave packet by an extra effective force. After taking into account the Berry curvature in the wave vector space, the trace of a free wave packet in the real space should be curved, feinting to break the Newton’s first law.

Since the discovery of quantum Hall effect^1^,^2^,^3^, physicists have established a new concept to characterize an exotic set of materials, called topological insulator, in physics and material science^4^,^5^,^6^. In such insulators, the electronic bands below the Fermi energy have topological nontrivial structures which are specified by the numbers called topological indices^7^. In experiments, several classes of materials have been proved to be topological insulators. But the proposed quantized transmission spectrum in these materials are seldom reported because the transport though edges is usually rumored by the finite conductance from the bulk states^8^,^9^,^10^.

It will be plausible to find similar topological phenomena in systems “cleaner” than the electronic ones. There are both theoretical and experimental works to extend the theory to photonic crystallines^11^,^12^,^13^,^14^,^15^,^16^,^17^, phonon in solids^18^,^19^, exciton systems^20^,^21^, electric circuits^22^,^23^, harmonic vibrational lattices^24^,^25^,^26^,^27^,^28^, floquet classical system^29^, and so on. The last three categories are based on classical mechanics and do not pose difficulties on the nano-sized material science and may allow observers to watch the chiral or the helical edge states by eyes^27^,^28^.

However the effect of unavoidable dissipations in the classical models has not been systematically investigated yet. In this paper, we try to solve this problem based on a 2-dimensional (2D) topological mechanical lattice. This investigation extends our understanding on these systems in two aspects. The first one is that our discussions are not based on a Hamiltonian representation because in fact there is no Hamiltonian for such systems. So it is impossible to borrow the discussions on the language of Hamiltonian from its quantum counterpart directly. The second one is that the symmetry which protects edge states should be reexamined in the presence of the dissipation. It is believed that the dissipation will induce decoherence and dephasing in quantum systems^30^,^31^,^32^,^33^,^34^,^35^,^36^. For instance, in the quantum Hall system, it will fuzz up the universal values of the Hall plateaus. On the contrary, we will show that in the classical systems the dissipation will not break the gauge invariance. This makes the chiral edge states robust against the strong dissipation. We will perform both gauge argument and numerical calculation to confirm this conclusion.

Furthermore, we find that the dissipation will induce a damping spectrum. Such damping dispersion can generate an effective force for a free moving wave packet, which seems to break the Newton’s first law at the first glance. We can understand this effect by considering a free wave packet whose profile in the wave vector space is a Gaussian function, 
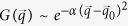
. In a dissipativeless system without external force, this profile does not alter with time so that the averaged wave vector 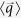 is a constant, 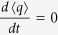. But in the dissipative system, the damping rate is a function of wave vector 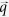, called the dispersion of damping. As a result, the profile is varying with time although the wave vector *q* is still good quantum number. Effectively, this variance can be expressed as an extra force acting on the wave packet, 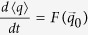. Such force and the nonzero Berry curvature will bend the trace of the wave packet, which can be observed in experiments.

The paper is organized as the following: In section 2, we present a topological mechanical system on a rotating square lattice. All our arguments will base on this model. In section 3, we introduce how to calculate the Chern number by the evolution of the centers of the Wannier functions. The gauge invariance that protecting the chiral edge states is discussed. We further illustrate that such gauge argument is still available in a disordered lattice. In section 4, we derive the equation of motion for a free wave packet. The dissipation induced force is discussed. Section 5 is for the conclusions and outlooks.

## The Model: A Rotating Square Lattice

**Figure 1:**
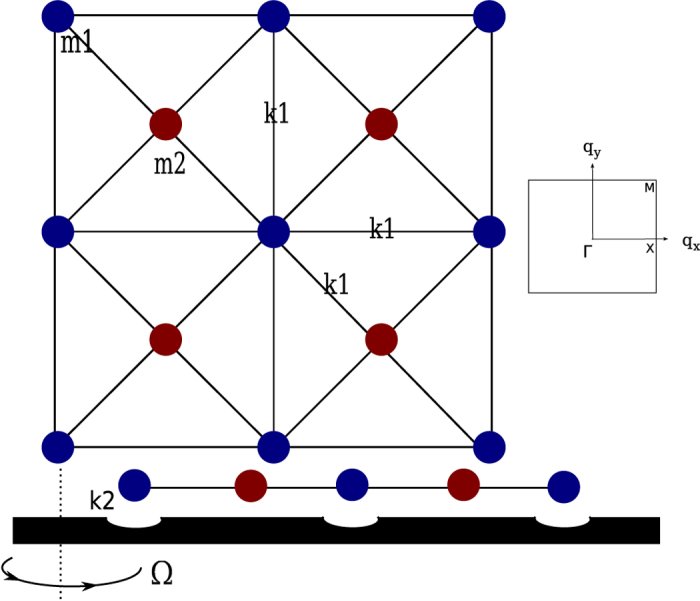
The square phononic lattice composed of MPs and springs, with top view (upper panel) and side view (lower panel). Each unit cell has two MPs with masses *m*_1_ and *m*_2_ respectively. The dark lines represent the springs with the spring constant *k*_1_. The lattice is mounted on a rotating plane whose angular velocity is Ω. Besides the upward supportive forces, the plain also interacts with MPs by restoring forces whose effective spring constant is *k*_2_ in the horizontal directions. We use the pits on the plane to represent such harmonic forces. Throughout this paper, we take *m*_1_ = *m*_2_ = *m* and *k*_2_ = 0.2*k*_1_. The first Brillouin zone is shown on the right. Here the lattice constant is taken as the length unit.

We first consider a square lattice shown in Fig. 1. Each unit cell has two inequivalent mass points (MPs) and each MP has two degrees of freedom, *R*_*x*_ and *R*_*y*_ in the plane. After Fourier transformation to the wave vector space 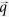, the vibrational motions are determined by such Newton’s equations:


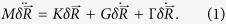


Here 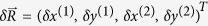 is the vector with the components representing the displacements of MPs, 1 and 2, away from their rest positions along *x* and *y* directions for the Bloch wave. The three terms on the right hand side stand for the restoring forces, the Coriolis forces and the dissipative forces, respectively.

The matrix *M* is


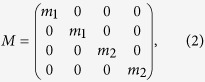


with *m*_1_ and *m*_2_ are the masses of the two MPs in each unit cell. The restoring force matrix *K* reads


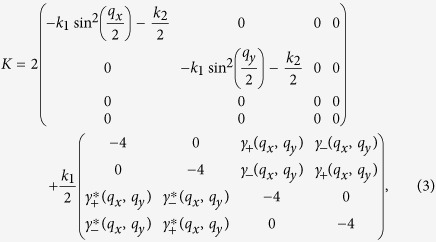


where *k*_1_ and *k*_2_ are the spring constants shown in Fig. 1, 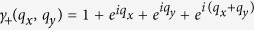 and 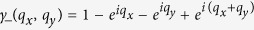, and *q*_*x*_, *q*_*y*_ are the wave vectors of the phonon modes along *x* and *y* directions,respectively. It is derived from the harmonic potential: 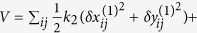

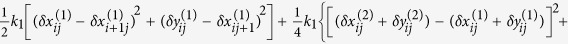

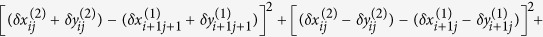

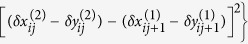
, where the subscripts *i* and *j* label the positions of a unit cell in the *x* and *y* directions.

*G* is the matrix describing the Coriolis force acting on the lattice,


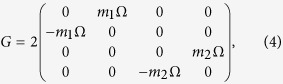


where Ω is the angular velocity of the plane with the positive direction defined in Fig. 1. The stationary centrifugal forces do not appear in this equation because we have taken the variations 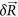 with respect to their new stationary positions where the centrifugal forces are compensated by the restoring forces of the springs. One may worry about the transverse motion because the spring is stretched in this case. But as the whole lattice is placed abaxial and the lattice constant is small, each mass point will fell similar centrifugal force. In this case, some of strings are stretched and some are compressed. So totally, they produce zero net force in the transverse direction. The centrifugal forces will also induce forces in the radial direction when the MPs are away from their new stationary positions. We also ignore them in the case of small Ω because their magnitudes are proportional to Ω^2^. For the dissipation term, we take isotropic dissipative forces with Γ = *γI*, where *I* is a 4 × 4 unit matrix.

Such second order differential equations can be solved as an eigenvalue problem by introducing new variables, 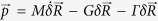. The Eq. 1 is rewritten as


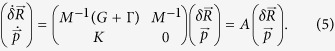


As 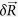, as well as 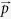, is varying with time as ~*e*^*iωt*^ for an eigenstate, the phonon dispersion will be obtained by solving the eigenvalue problem: *det*(*iωI* − *A*) = 0, where *A* is the matrix on the right hand side of Eq. 5.

**Figure 2:**
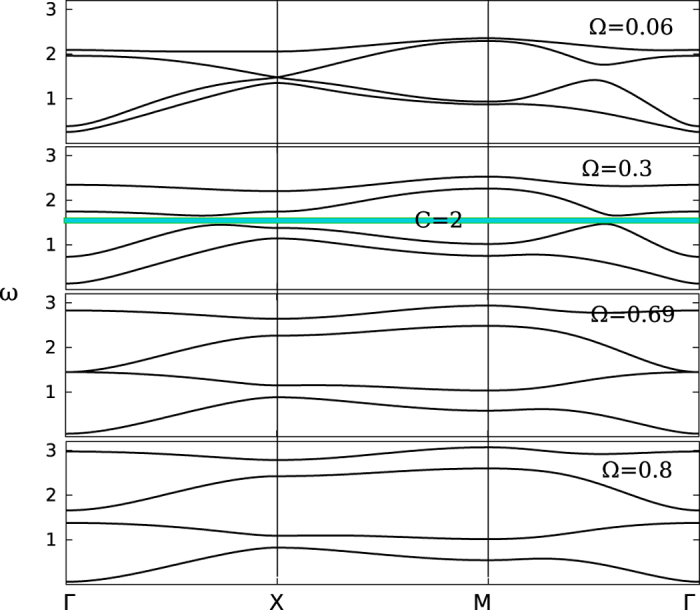
The phonon dispersion for the dissipativeless model. The angular velocity is taken at Ω = 0.06, 0.3, 0.69 and 0.8 in the panels from up to down. The model is in a topological phase when 0.06 < Ω < 0.69. We illustrate the topologically nontrivial gap with a green box and indicate its Chern number *C* = 2 there explicitly. All other gaps are topological trivial.

In the absence of dissipation, the lattice has a mechanical topological phase. In Fig. 2, we show the phonon dispersion at several Ω. The parameters are taken as *m*_1_ = *m*_2_ = *m*, *k*_2_ = 0.2*k*_1_ and *γ* = 0. Throughout this paper, all physical quantities are taken as dimensionless. It is easy to recast the unit at the end of the calculations (for instance the eigen-frequencies *ω* and the angular velocity Ω are both taken in the units of 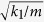). This model has a topologically nontrivial phase when 0.06 < Ω < 0.69. We explicitly denote the topologically nontrivial gap corresponding to a Chern number, *C* = 2, by the green box in the figure. All other gaps are topologically trivial.

## The Chern Number in the Present of Dissipation

Now we discuss how to calculate the Chern number in the presence of dissipation. At the very beginning, a definition of inner product for the eigenstates must be discussed. It has been noticed that although the topological mechanism is considered as a classical mimic of the Chern insulator, there is still a technical difference between them. In the last section, we have shown that the motion of the classical lattice follows the Newton’s law and the vibrational eigenstates are obtained from a non-hermitian matrix *A*. So the eigenstates with different eigenvalues are not orthogonal to each other as usual. Such technical problem has been solved by a redefinition of inner product for the eigenstates^19^,^24^. But the problem becomes worse as the dissipation is included in the system. The inner product in ref. 19, 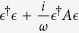, fails because there is no Hamiltonian for a dissipative system. While the definition of inner product in ref. 24, 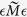, becomes non-orthogonal again. Here 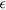 is the polarization vector, *A* is the effective vector potential in the Hamiltonian and 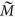 is the effective mass matrix. In this paper, the inner product of the eigenstates is redefined by the left and right eigenstates directly. We denote the state in the bra-ket notation with 〈*u*_*a*_| and |*u*_*a*_〉, which are the left and the right *a*th eigenstates of the matrix *A* respectively. For the case *a* ≠ *b*, it is easy to find that 〈*u*_*a*_|*u*_*b*_〉 = 0. One should note that, in the new definition, the states include both the components of the position variables 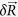 and the auxiliary variables 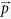.

Now we describe how to calculate the Chern number by studying the evolution of the centers of Wannier functions. It is extended from the numerical method developed for electronic topological insulators. Here we first briefly review this method in the electronic system. The Chern number is expressed as^37^,^38^,


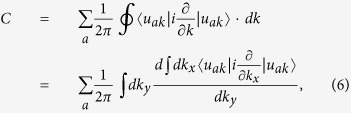


where the summation is over all the occupied bands and the closed path integration is along the boundaries of the first Brillouin zone (BZ). As 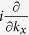 is the position operator in the *x* direction, the Chern number can be written down as 
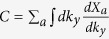
, where 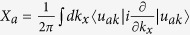 is the position (in the *x* direction) of the Wannier functions for the *a*th band. Yu *et al.* have developed a numerical method to calculate 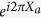 instead of *X*_*a*_^37^. Its physical meaning is to calculate the accumulated phase for the *a*th band when changing *k*_*x*_ by one reciprocal vector in the BZ. One can divide such variation into *M* pieces, and each piece is a nonzero matrix only for 
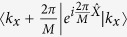
, in the momentum space. 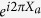 will be obtained from the eigenvalues of a product matrix by multiplying all these nonzero matrices. This numerical method has been applied to investigate the topologically nontrivial electronic bands^39^,^40^,^41^.

In our classical system, the Chern number can still be considered as the total displacement of the Wannier functions (in the *x* direction) for the studied phonon bands as changing *q*_*y*_ by one reciprocal vector. But the inner product for |*u*_*aq*_〉 and 〈*u*_*aq*_| must take the form of the new definition. As a result, the project operator inserted into the pieces 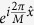 should be changed to 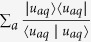. So the total phase Φ(*q*_*y*_) for the interesting *n* phonon bands is 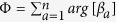, where *β*_*a*_ is the eigenvalues of the *n* × *n* matrix 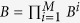 with


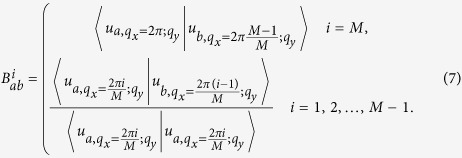


Here 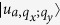 and 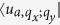 are the right and left *a*th eigenvectors of the matrix *A* in Eq. 5 and *n* is the number of bands below the interesting gap.

In this model, because the topologically nontrivial gap is in between the second and the third phonon bands, the matrix *B*^*i*^ in the above expression is a 2 × 2 matrix with *a* and *b* run over the indeces of phonon bands, 1 and 2.

**Figure 3:**
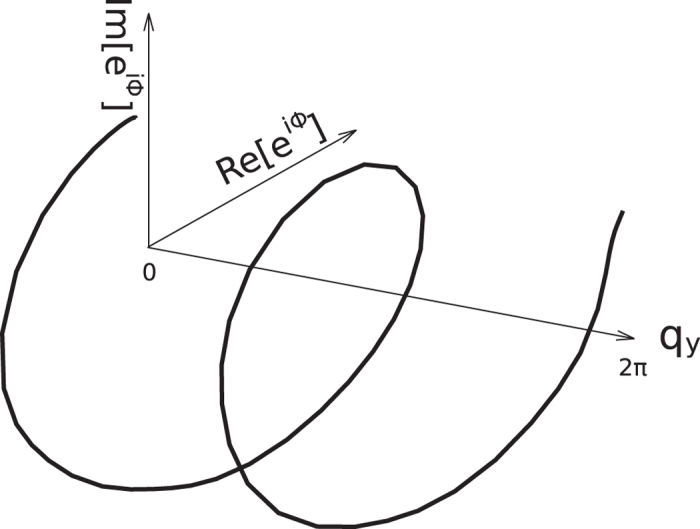
The total phase Φ in 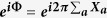 as *q*_*y*_ is varying 2*π*. The angular velocity is Ω = 0.3 and the strength of dissipation is *γ* = 0.6. 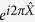 is divided into *M* = 10 pieces in the calculation.

In Fig. 3, we show how the total phase in 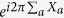 changes as *q*_*y*_ is varying 2*π*. Here the angular speed is Ω = 0.3 and the dissipative strength is *γ* = 0.6. We see that the Wannier function centers move totally 2 unit cells as *q*_*y*_ is varying one reciprocal vector. So we can conclude that the Chern number *C* is 2 for the gap between the second and the third bands.

As we expected, dissipation will not alter the Chern number as long as the band gap is not closed. Now we employ a *N*_*x*_ × *N*_*y*_ lattice in the cylinder geometry and discuss the inevitable chiral edge states just like Laughlin did in quantum Hall system^3^. In such a classical system, there is neither Fermi energy nor Fermi-Dirac distribution for the eigenstates. But as the eigenvalue spectrum is an intrinsic property and is independent of the number of the excitations in the system, we can assume a special situation in which all the eigenstates below the gap are excited and the states above that are empty. Now supposing there is a gauge transformation that continously changing 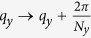, for the wave vector in the azimuthal direction. As the states in an individual band are all excited or all empty for *q*_*y*_s, such gauge transformation is equivalent to a change of one *q*_*y*_ by 2*π*. From the evolution of the Wannier centers in the cylindrical direction as varying *q*_*y*_, two excited Wannier states have been pumped from the left edge to the right edge. This gauge transformation is imitating the magnetic flux quanta penetrating the center of a cylinder in electronic quantum Hall system. As the variance of *q*_*y*_ by one reciprocal vector changes nothing, the gauge transformation is invariant and the system must come back to its initial state after the transformation. To compensate the pumped bulk states, there must have edge states at the boundaries of the cylinder. Such edge states will connect the low excited bands to the high empty bands twice during the pump. According to this argument, the existence of the chiral gapless edge states in the spectrum is concluded and such edge states must be robust against strong dissipation.

**Figure 4:**
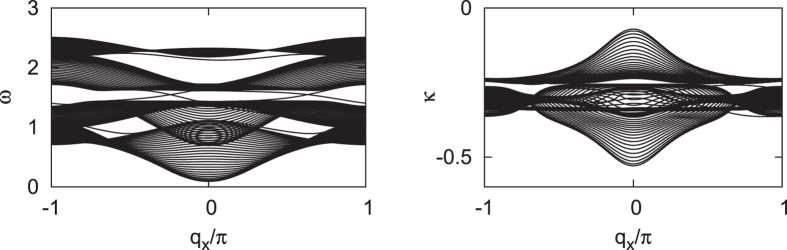
The real parts (left) and the imaginary parts (right) of the eigen-frequencies for a ribbon with width *W* = 30. The two chiral edge modes with finite life time are confirmed.

We confirm the above conclusion by a calculation of spectrum of a ribbon with two geometric boundaries. In the left panel of Fig. 4, we show the real parts of the eigenvalues of the ribbon with width *W* = 30. All parameters are the same as those in Fig. 3. An open boundary condition is taken in the transverse direction and the wave vector *q*_*x*_ along the longitudinal direction is a good quantum number. We can see two chiral edge modes whose eigen-frequencies are within the gap between the second and the third phonon bands. Interestingly, this gap is more pronounced in the dissipative case than that in the dissipativeless case. The dissipation induced damping, which corresponds to the imaginary parts of the eigenvalues, are shown in the right panel of Fig. 4. It can be seen that all eigenstates are damped, but the damping rate is not uniform for different eigenstates at different *q*_*x*_. This is the origin of the dissipation induced force that is discussed in the next section.

**Figure 5:**
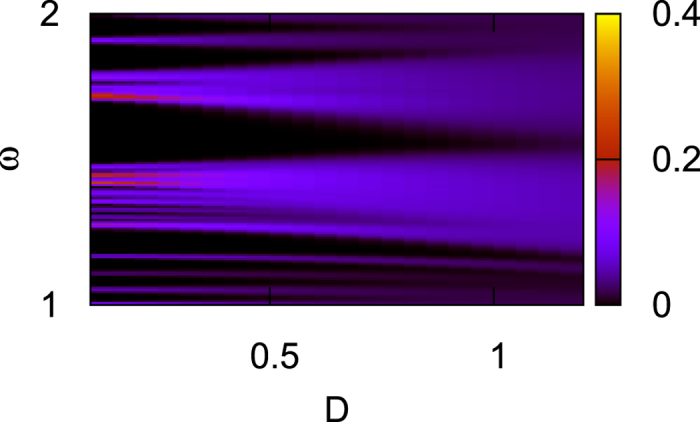
The averaged density of states (in arbitrary unit) as a function of the strength of disorder *D* and the frequency *ω*. The gap (at *ω* ~ 1.5) between the bands 2 and 3 is closed around *D* = 0.9.

At the end of this section, we show that the above gauge argument can be extended to the disordered case. In Fig. 5, we show the averaged density of states for the disordered *N* × *N* phononic lattice. Here *N* = 10 and the average is over 2000 samples. The spring constant *k*_1_ is randomly chosen in the range [(1 − *D*/2)*k*_1_, (1 + *D*/2)*k*_1_]. Other parameters are fixed and are the same as those in Fig. 3. It is found that the gap between the second band and the third band is not closed until the strength of disorder reaches *D* = 0.9. When the gap is not closed, the gauge argument is performed on an ensemble of cylinders with different disordered configurations. The number of pumped bulk states can still be investigated by the evolution of the centers of the Wannier functions. Similar to that in the electronic system^42^, this evolution can be calculated with the present method on the ensemble of super-lattices in which *N* × *N* disordered samples are taken as the unit cell. In this case, the first and the second bands split into totally 2*N*^2^ bands and the total phase Φ is calculated for these bands. We find that the Chern number *C*, which characterizes the evolution of the Wannier functions, is fixed at 2 in the ensemble. It will fluctuate from sample to sample only when the gap is closed by the disorder for *D* > 0.9. So from this gauge investigation, it is concluded that the chiral edge states in the topological mechanical system are robust against disorder and dissipation. This provides further support for the conclusions of Fig. 3 in ref. 24, where the topological propagation immune against disorder is shown. We have also studied the randomness of the mass instead of the strength of springs and found similar results.

## Dissipation Induced Force and its Effect on the Motion of Wave Packet

Supposed that there is a free wave packet composed by the states in the *a*th phonon band. Its profile at the initial time *t* = 0 is 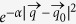 in the wave vector space. This initial state is similar to the states in the discussion of semi-classical equation of motion for quasiparticles in solid.

As there is no external force, 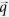 is a good quantum number. We can write down the wave packet in the real space at any time *t* easily,


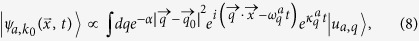


where 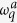 and 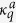 are the real and imaginary parts of the eigen-frequency for the *a*th eigenstate |*u*_*a*,*q*_〉 at the wave vector 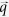. After expanding 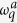, 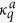 and |*u*_*a*,*q*_〉 to the first order of the derivation of 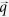 at 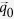, we rewrite the above equation as


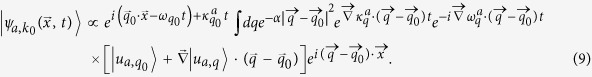


One can simplify 
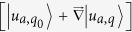
 with the berry phase 
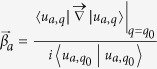
, to 
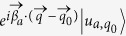
. After substituting 
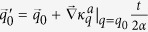
 and 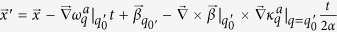 into the integration and assuming that *t* is within a small time interval, the wave packet becomes


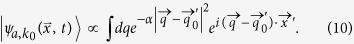


So the motion of wave packet satisfies the following equations of motion for its centers 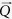 and 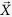 in the wave vector and real spaces, respectively:


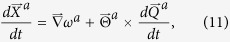



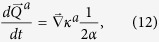


where 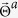 is the berry curvature. Compared with those of the dissipationless case, we find that the dissipation will induce an effective force whose amplitude is proportional to the gradient of the damping rate and inversely proportional to the size of the wave packet in the real space.

**Figure 6:**
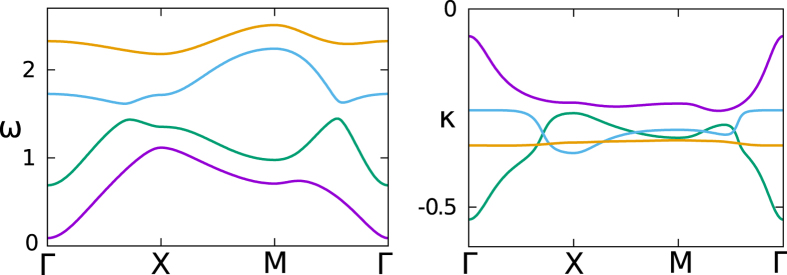
The dispersion of phonon eigen-frequency *ω* (left) and damping rate *κ* (right) along the high symmetric directions in BZ.

In Fig. 6, we show the dispersions of the real and imaginary parts, *ω* and *κ*, of the eigen-frequencies for the dissipative model. It seems that the damping rate is strongly dispersed in this topological system. This dispersion comes partially from the finite value of Ω. It is also caused by the fact that the damping of a vibrational mode is proportional to its velocity. As the time derivation of the displacement of an eigen-mode will generate a multiplier, the eigen-frequency *ω*, to the velocity, the eigen-modes with different eigen-frequencies will suffer different damping rates.

**Figure 7:**
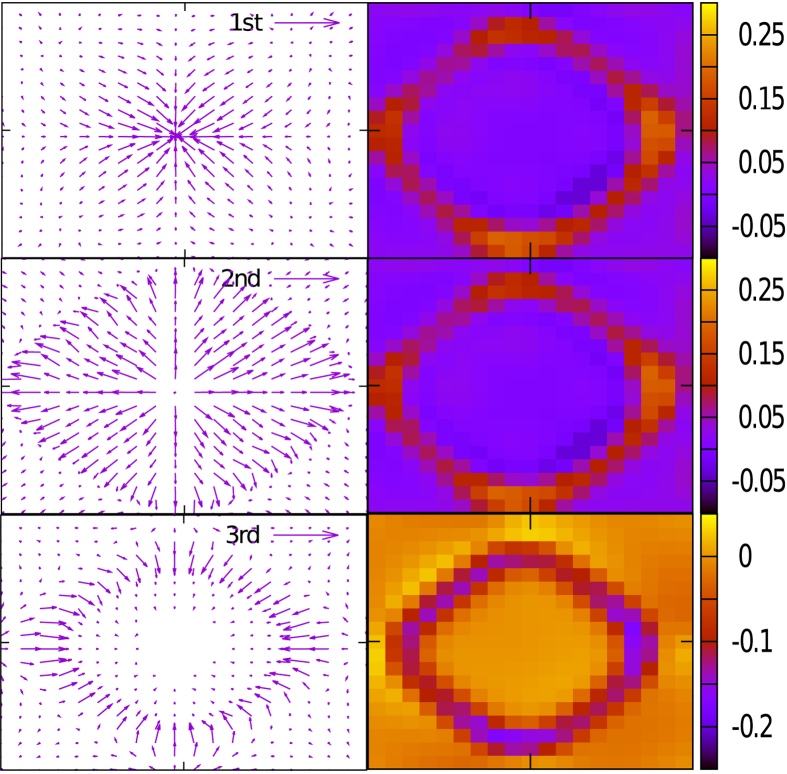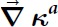 (left) and the strength of berry curvature 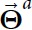 perpendicular to the BZ plane (right) are shown for the first three bands, *a* = 1, 2, 3 from up to down.

In Fig. 7, we plot the gradient of *κ*, 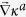 in the BZ with small arrows in the left panels and the strength of Berry curvature 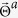 with the contour in the right panels. The Berry curvature 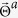 is calculated by discretizing the BZ into small grids and counting the accumulated phase by the eigenstates along the edges of each grid^24^. Of course, the new definition of inner product must be taken in the calculation. Here only those for the first three phonon bands are plotted and the forth band is ignored because, as shown in Fig. 6, *κ* is not varying rapidly for this band. It is shown that for the 2nd and the 3rd bands, there is a cirque in the BZ, in which both 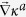 and 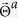 are relatively large. According to the equations of motion, 
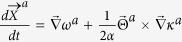
, the free wave packet composed by the eigenstates of the these bands suffers a tendency towards the transverse direction caused by the combination of Berry curvature and the extra force in the last term. As this term is also varying with 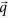, as well as with the time, the trace of the wave packet is a curved line, similar to the trace of electron in 2D hall bar with longitudinal electric field. We suggest that such effect may be observed by preparing the proper wave packet composited by the states for which both 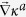 and 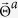 are large in the 2nd band or in the 3rd bands.

## Conclusions and Outlooks

The chiral edge states in the topological mechanical system are robust against dissipation and disorder. We also find that the dissipation can induce a dispersion of damping for the eigenstates. As a result, an extra force appears in describing the motion of free wave packet. After taking into account the non-zero Berry curvature in such system, we find that the free wave packet will swerve even through there is no external force acting on the packet. One should be aware that the Coriolis forces and dissipative forces have been taken into account during the calculation of bands so that they should not be considered as external forces again during the calculation for the wave packet. We also suggest that such effect should also exist in dissipative photonic system. Besides this, a general method to calculate the Chern numbers in such systems is developed and we believe that it is also applicable in non-hermitian quantum systems.

## Additional Information

**How to cite this article**: Xiong, Y. *et al.* The effects of dissipation on topological mechanical systems. *Sci. Rep.* **6**, 32572; doi: 10.1038/srep32572 (2016).
